# Oral manifestations associated with long COVID: a scoping review

**DOI:** 10.1590/0034-7167-2025-0198

**Published:** 2026-07-06

**Authors:** Emylly Lopes Theodoro, Karina Marques Prediger, Marielle Aparecida Damacena, Camilly Victória da Silva, Rodrigo das Neves Cano, Silvia Carla da Silva Andre Uehara

**Affiliations:** I Universidade Federal de São Carlos. São Carlos, São Paulo, Brazil

**Keywords:** Oral Manifestations, Mouth Diseases, Post-Acute COVID-19 Syndrome, Periodontal Diseases., Manifestaciones Bucales, Enfermedades de la Boca, Síndrome Post Agudo de COVID-19, Enfermedades Periodontales.

## Abstract

**Objectives:**

to map scientific evidence on oral manifestations originating during long COVID.

**Methods:**

this is a scoping review based on the method described by JBI. Primary articles published in Portuguese, English, and Spanish between March 2020 and December 2024 were included from the PubMed, Web of Science, Virtual Health Library, Scopus, Excerpta Medica dataBASE, and Scientific Electronic Library Online databases, and a descriptive analysis was performed.

**Results:**

of the 15 studies analyzed, the most frequent oral manifestations of long COVID were taste alterations, xerostomia, difficulty chewing, gingival bleeding, periodontitis, and changes in the teeth.

**Conclusions:**

an association between long COVID and various oral manifestations was evidenced, impacting the quality of life of patients. Factors such as age, comorbidities, and social inequalities influence the persistence of these manifestations, with a higher prevalence in women. Multiprofessional collaboration and clearer guidelines are essential to improve dental care.

## RESUMEN

**Objetivos:**

mapear la evidencia científica sobre manifestaciones orales surgidas durante la COVID-19 prolongado.

**Métodos:**

se realizó revisión exploratoria basada en la metodología descrita por JBI. Se incluyeron artículos originales en portugués, inglés y español, publicados entre marzo de 2020 y diciembre de 2024, indexados en PubMed, *Web of Science*, Biblioteca Virtual en Salud, Scopus, *Excerpta Medica dataBASE* y *Scientific Electronic Library Online*. Se llevó a cabo un análisis descriptivo.

**Resultados:**

de los 15 estudios analizados, las manifestaciones orales más frecuentes del COVID prolongado fueron alteraciones del gusto, xerostomía, dificultad para masticar, sangrado gingival, periodontitis y cambios en los dientes.

**Conclusiones:**

el COVID-19 prolongado se asoció con diversas manifestaciones orales que afectaron la calidad de vida de los pacientes. Factores como la edad, las comorbilidades y las desigualdades sociales influyen en la persistencia de estas manifestaciones, con una mayor prevalencia en mujeres. La acción multidisciplinaria y la implementación de guías clínicas más claras son esenciales para mejorar la atención odontológica.

## INTRODUCTION

Long COVID has been characterized by the World Health Organization as the manifestation of symptoms that generally appear within three months of acute COVID-19 infection, lasting for at least two months, and cannot be explained by another diagnosis^(1)^. In Brazil, the term “post-COVID conditions” was officially adopted, defined as the persistence or emergence of signs, symptoms, and/or clinical changes starting four weeks after the initial infection, which may vary over time, with possible relapses and, in some cases, progression to serious complications^(2)^. In this study, the term “long COVID” was chosen, in accordance with the predominant terminology in the international literature.

Since the beginning of the pandemic, it has been observed that COVID-19 goes beyond an acute respiratory infection, affecting different body systems. In the post-acute phase, the most frequently reported persistent symptoms include fatigue, dyspnea, chest pain, myalgia, cognitive disorders, and sleep disturbances, varying in frequency and intensity among individuals^(3,4)^. These manifestations have had significant impacts on patients’ quality of life and functional capacity, even many months after recovery from the initial infection^(5)^.

The diversity of clinical presentations of long COVID is associated with factors such as age, sex, presence of comorbidities, severity of the initial illness, need for hospitalization or admission to an Intensive Care Unit (ICU), in addition to vaccination status⁽^6,7^⁾. Evidence suggests that women and unvaccinated individuals are at higher risk of developing persistent symptoms^(8)^. Exaggerated immune response and prolonged inflammatory changes have also been pointed to as possible pathophysiological mechanisms that explain this multifactorial condition^(9)^.

In this scenario, the oral cavity emerges as a site of interest for investigating the clinical manifestations of long COVID. Tissues such as the oral mucosa, tongue, and salivary glands express high levels of angiotensin-converting enzyme 2, which acts as the main receptor for the entry of the virus into human cells^(10)^. This biological characteristic suggests that the mouth may be not only an entry point for the virus but also a region vulnerable to persistent clinical manifestations. It is important to note that the terminology “oral manifestations” was adopted in this study because it refers to disorders of the mouth that accompany diseases or lesions that are not of oral origin, as is the case with long COVID^(11)^.

Studies on oral symptoms of long COVID remain fragmented, with isolated reports and a lack of comprehensive reviews that systematize the existing findings. Therefore, given the impact of long COVID and the manifestation of oral symptoms in this condition, it is essential to map and synthesize the available evidence. Identifying these manifestations can facilitate the implementation and improvement of multidisciplinary follow-up protocols for these patients.

## OBJECTIVES

To map scientific evidence on oral manifestations originating during long COVID.

## METHODS

### Ethical aspects

As this is a review study, approval from the Research Ethics Committee was not required. However, it was ensured that all primary studies included in this review complied with current ethical standards.

### Study design

This is a scoping review, which was conducted following the methodological stages described by JBI^(12)^, adhering to the Preferred Reporting Items for Systematic reviews and Meta-Analyses extension for Scoping Review guidelines^(13)^.

### Methodological procedure

To construct the guiding question, the PCC strategy was applied, which represents a mnemonic for P (Population) (People with long COVID), C (Concept) (oral manifestations), and C (Context) (COVID-19 pandemic), to be defined as: what are the main oral manifestations presented by people with long COVID?

Primary studies published in Portuguese, English, and Spanish between March 2020 and December 2024 were included. Articles whose titles and abstracts did not fall within the scope of the research, as well as opinion articles, editorials, reviews, theses, dissertations, and secondary reviews, were excluded. This decision is based on the purpose of concentrating the review on original studies published in peer-reviewed scientific journals, ensuring greater methodological rigor, reliability of evidence, and homogeneity of the sources analyzed. Additionally, filters for publication period, language, and document type were applied in each database, according to the availability of the consulted platforms. The reference lists of all studies found were also checked.

The protocol for this scoping review was registered in the Open Science Framework (OSF), under DOI 10.17605/OSF.IO/R6UZ8, and is available at: https://osf.io/r6uz8.

### Data collection, organization, and analysis

The search for articles was conducted in the electronic databases PubMed, Web of Science, Virtual Health Library, Scopus, Excerpta Medica dataBASE, and Scientific Electronic Library Online on January 17, 2025. Initially, an exploratory search was performed to identify the different ways the phenomenon is indexed in the databases (controlled descriptors Medical Subject Headings (MeSH)/Health Sciences Descriptors (In Portuguese, *Descritores em Ciências da Saúde* - DeCS) and free keywords), in order to include relevant terminological variations and synonyms.

The searches were conducted using descriptors and their synonyms, which are listed in DeCS and MeSH, including: Long COVID; Post-Acute COVID-19 Syndrome; Post-COVID Conditions; Signs and Symptoms; Oral Manifestations; Periodontal Diseases; Periodontosis; Periodontal Atrophy; Gingivo-Osseous Atrophies; Stomatognathic Diseases; Dental Diseases; Mouth and Tooth Diseases; Oral Pathology; Oral and Maxillofacial Pathology; Tooth Loss. The combination of descriptors was performed using the Boolean operators OR and AND (Chart 1).

**Chart 1:** Search strategies used in the databases, São Carlos, São Paulo, Brazil, 2025

Database	Reference	Total articles
PubMed	((((((((((((((“Long COVID”[Title]) OR “Post-Acute COVID-19 Syndrome”[Title]) OR “Post-COVID Conditions”[Title]) AND (“Signs[Title] AND Symptoms”[Title])) OR “Oral Manifestations”[Title]) OR “Periodontal Diseases”[Title]) OR “Parodontosis”[Title]) OR “Periodontal Atrophy”[Title]) OR “Gingivo-Osseous Atrophies”[Title]) OR “Stomatognathic Diseases”[Title]) OR “Dental Diseases”[Title]) OR (“Mouth[Title] AND Tooth Diseases”[Title])) OR “Pathology, Oral”[Title]) OR (“Oral[Title] AND Maxillofacial Pathology”[Title])) OR “Tooth Loss”[Title]	573
Virtual Health Library	(ab:(“Long COVID” OR “Post-Acute COVID-19 Syndrome” OR “Post-COVID Conditions” OR “*Síndrome de COVID-19 Pós-Aguda*” OR “*Síndrome Post Agudo de COVID-19*”)) AND (ab:(“Signs and Symptoms” OR “*Sinais e Sintomas*” OR “*Signos y Síntomas*”)) AND (ab:(“Oral Manifestations” OR “*Manifestações Bucais*” OR “*Manifestaciones Bucales*”)) OR (ab:(“Periodontal Diseases” OR Parodontosis OR “*Doenças Periodontais*” OR “*Enfermedades Periodontales*”)) OR (ab:(“Periodontal Atrophy” OR “Gingivo-Osseous Atrophies” OR “*Atrofia Periodontal*”)) OR (ab:(“Stomatognathic Diseases” OR “Dental Diseases” OR “Mouth and Tooth Diseases” OR *“Doenças Estomatognáticas*” OR “*Enfermedades Estomatognáticas*”)) OR (ab:(“Pathology, Oral” OR “Oral and Maxillofacial Pathology” OR “*Patologia Bucal*” OR “*Patología Bucal*”)) OR (ab:(“Tooth Loss” OR “*Perda de Dente*” OR “*Pérdida de Diente*”))	95
Scopus	( ABS ( “Long COVID” ) OR ABS ( “Post-Acute COVID-19 Syndrome” ) OR ABS ( “Post-COVID Conditions” ) AND ABS ( “Signs and Symptoms” ) OR ABS ( “Oral Manifestations” ) OR ABS ( “Periodontal Diseases” ) OR ABS ( parodontosis ) OR ABS ( “Periodontal Atrophy” ) OR ABS ( “Gingivo-Osseous Atrophies” ) OR ABS ( “Stomatognathic Diseases” ) OR ABS ( “Dental Diseases” ) OR ABS ( “Mouth and Tooth Diseases” ) OR ABS ( “Pathology, Oral” ) OR ABS ( “Oral and Maxillofacial Pathology” ) OR ABS ( “Tooth Loss” ) )	95
Scientific Electronic Library Online	BY TITLE: (ti:(^*^”Long COVID” OR “Post-Acute COVID-19 Syndrome” OR “Post-COVID Conditions” OR “*Síndrome de COVID-19 Pós-Aguda*” OR “*Síndrome Post Agudo de COVID-19*” )) AND (ti:(“Signs and Symptoms” OR “*Sinais e Sintomas*” OR “*Signos y Síntomas*” )) AND (ti:(“Oral Manifestations” OR “*Manifestações Bucais*” OR “*Manifestaciones Bucales*” )) OR (ti:(“Periodontal Diseases” OR Parodontosis OR “*Doenças Periodontais*” OR “*Enfermedades Periodontales*” )) OR (ti:(“Periodontal Atrophy” OR “Gingivo-Osseous Atrophies” OR “*Atrofia Periodontal*” )) OR (ti:(“Stomatognathic Diseases” OR “Dental Diseases” OR “Mouth and Tooth Diseases” OR “*Doenças Estomatognáticas*” OR “*Enfermedades Estomatognáticas*” )) OR (ti:(“Pathology, Oral” OR “Oral and Maxillofacial Pathology” OR “*Patologia Bucal*” OR “*Patología Bucal*”)) OR (ti:(“Tooth Loss” OR “*Perda de Dente*” OR “*Pérdida de Diente*”))	40
Web of Science	(((((((TI=(“Long COVID” OR “Post-Acute COVID-19 Syndrome” OR “Post-COVID Conditions”)) AND TI=(“Signs and Symptoms”)) AND TI=(“Oral Manifestations”)) OR TI=(“Periodontal Diseases” OR “Parodontosis”)) OR TI=(“Periodontal Atrophy” OR “Gingivo-Osseous Atrophies” )) OR TI=(“Stomatognathic Diseases” OR “Dental Diseases” OR “Mouth and Tooth Diseases”)) OR TI=(“Pathology, Oral” OR “Oral and Maxillofacial Pathology”)) OR TI=(“Tooth Loss”)	888
Excerpta Medica dataBASE	((‘long Covid’:ti OR ‘post-acute Covid-19 syndrome’:ti OR ‘post-Covid conditions’:ti) AND ‘signs and symptoms’:ti AND ‘oral manifestations’:ti OR ‘periodontal diseases’:ti OR ‘parodontosis’:ti OR ‘periodontal atrophy’:ti OR ‘gingivo-osseous atrophies’:ti OR ‘stomatognathic diseases’:ti OR ‘dental diseases’:ti OR ‘mouth and tooth diseases’:ti OR ‘pathology, oral’:ti OR ‘oral and maxillofacial pathology’:ti OR ‘tooth loss’:ti) AND [2020-2024]/py	927

For study selection, after implementing the search strategy in each database, the references were exported to the StArt (State of the Art through Systematic Review) web application for study selection at two levels. At this stage, duplicate entries were automatically excluded by the software, and subsequently, a manual verification was performed for confirmation. The initial selection was carried out by reading titles and abstracts, followed by reading the full article. The StArt review tool was developed by the Software Engineering Research Laboratory at *Universidade Federal de São Carlos*.

Eligible studies were assessed in full by two researchers independently, and any discrepancies would be discussed with a third researcher and resolved by consensus. However, it was not necessary to involve a third researcher, as there were no discrepancies. Data were extracted from each included article, such as authors, country where the study was conducted, publication date, research objective, study design, and main results. Charts and descriptions were used to present the results, in order to answer the guiding question of the review.

## RESULTS

**Figure 1:**
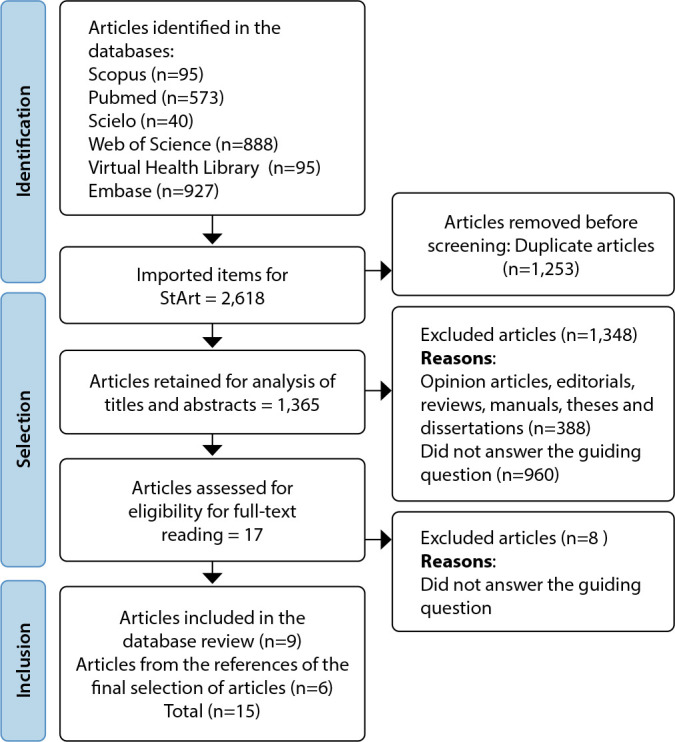
Flowchart for article selection, São Carlos, São Paulo, Brazil, 2025

A total of 2,618 articles were found in the databases, of which 1,253 were removed due to being duplicates. Of the 1,365 studies analyzed, 388 were excluded for being opinion articles, editorials, reviews, manuals, theses, or dissertations, according to the previously established exclusion criteria, and 960 after reading the title and abstract. Thus, 17 were read in full, with eight being excluded for not answering the guiding question, and nine selected for the study. Analyzing the references of the nine selected articles, six studies were included from the cited references because they addressed the proposed topic, totaling 15 studies in this review (Figure 1).

Among the 15 articles included, three (20%) were conducted in India, two (13.3%) in Iran, and one (6.7%) in each of the following countries: Sweden, Poland, United Kingdom, Iraq, Saudi Arabia, Brazil, France, Spain, Indonesia, and the Faroe Islands. It is observed that 14 (93.3%) articles were published in English, and only one (6.7%) in Portuguese.

Concerning study design, six (40%) were cross-sectional studies, five (33.3%) were cohort studies, two (13.3%) were retrospective studies, one (6.7%) was a longitudinal study, and one (6.7%) was a case report.

Included studies were described in terms of objective, study design, study population characterization, and main evidence found. It is noteworthy that, in the post-acute phase of SARS-CoV-2 infection, one or more oral manifestations were reported, including dental manifestations when they affect periodontal structures, as systematized in Chart 2.

**Chart 2:** Description of the articles according to author, year, location, objective, study design, sample, and main results, São Carlos, São Paulo, Brazil, 2025

Author, year, and place	Objective	Study design and sample (n)	Study population characterization	Main evidence found
Moradian S.T. *et al*., 2020, Iran^(14)^	Assess the frequency of late symptoms in patients with COVID-19.	Cross-sectional study, n=200	80% of men, with an average age of 55 years	Persistent taste disturbances were reported in 8% of patients six weeks after hospital discharge, indicating gustatory sequels of long COVID.
Asadi-Pooya A.A. *et al*., 2021, Iran^(15)^	Examine risk factors associated with the development of long COVID in a large cohort.	Retrospective study, n=4,681	52.9% of men, with an average age of 52 years	Loss of taste occurs in 3% of cases, more frequently in women, people with respiratory illnesses, and those with prolonged hospital stays.
Zayet S. *et al*., 2021, France^(16)^	Describe patients with post-COVID-19 syndrome with persistent symptoms and compare them to the control group.	Retrospective study, n=354	63% of women, aged between 19 and 98 years	31.5% reported loss of taste approximately nine months after infection, reinforcing the persistence of gustatory symptoms.
Riestra-Ayora J. *et al*., 2021, Spain^(17)^	Determine the incidence and evolution of olfactory and gustatory dysfunctions related to COVID-19.	Cohort study, n=320	81.9% of women, aged between 18 and 65 years 60.5% presented persistent taste symptoms.	60.5% presented persistent gustatory symptoms, with 12% experiencing no recovery, and 14.4% experiencing partial recovery after six months.
Petersen M.S. *et al*., 2021, Faroe Islands^(18)^	Describe acute and long-term COVID symptoms in non-hospitalized patients.	Longitudinal study, n=180	54% of women, with an average age of 39.9 years.	Loss of taste was reported in 16.4% during the acute phase, persisting in 1.7% in the late follow-up among non-hospitalized patients.
Rafałowicz B. *et al*., 2022, Poland^(19)^	Present, through six case studies, the most common oral symptoms of long COVID in patients with a history of SARS-CoV-2 infection.	Case report, n=6	Four men (66%) and two women (34%) between 43 and 72 years old	Clinical cases have shown dry mouth, oral lesions, tooth loss, and tongue changes associated with long COVID.
Alfadda A.A. *et al*., 2022, Saudi Arabia^(20)^	Investigate clinical and biochemical characteristics of patients six months after hospital discharge.	Cohort study, n=98	51 (52%) men, with an average age of 48 years	Loss of taste and appetite was reported by 7.1% six months after hospital discharge.
Muthyam A.K. *et al*., 2022, India^(21)^	Determine oral manifestations among patients recovered from COVID-19.	Cross-sectional study, n=100	51% of men and 54% of women >35 years old	Xerostomia (11%), ulcerations (12%), taste alterations (11%), gingival bleeding, and chewing difficulties were identified.
Johansson A.K. *et al*., 2023, Sweden^(22)^	Provide information on general and orofacial symptoms of acute and long COVID-19 in Swedish survivors, and to identify predictive/protective factors.	Cohort study, n=283	132 patients with long COVID, 60 women (45%) and 72 men (55%) between 80 and 90 years old	46% presented with oral symptoms, such as difficulty chewing, cracked teeth, dry mouth, eating difficulties, gum bleeding, dysphagia, taste alterations, and ulcers. Moreover, 14% had ≥5 symptoms. Persistent ageusia was observed for up to six months after recovery, although in decreasing proportions over time.
Al-Magsoosi M.J.N. *et al*., 2023, Iraq^(23)^	Determine the prevalence of oral manifestations of COVID-19 in recovered patients.	Cross-sectional study, n=574	378 (88.3%) women and 196 (11.7%) men, aged between 18 and 78 years	Oral manifestations occurred in 88.3% of cases, mainly ageusia, xerostomia, and dysphagia. Only ageusia persisted, with an association to severity and age, but no relation to sex, smoking, or comorbidities.
Araújo A.D.D.G. *et al*., 2023, Brazil^(24)^	Analyze factors related to long COVID in the adult population of Brazil.	Analytical cross-sectional study, n=288	71.9% of women, aged between 18 and 91 years	The clinical manifestations associated with persistence were fever, chills, headache, cough, anosmia, ageusia, dyspnea, and diarrhea. Among those with persistent symptoms, headache (24.65%), cough (21.88%), and fever (21.18%) were prevalent, while diarrhea and dyspnea occurred less frequently (13.19%).
Gujral H.S. *et al*., 2023, India^(25)^	Study long-term effects up to 12 months after acute COVID-19 and analyze risk predictors.	Cohort study, n=120	63.3% of men, with an average age of 41 years	3.3% experienced persistent loss of taste after hospitalization, regardless of admission to the Intensive Care Unit.
Nair C.V. *et al*., 2023, India^(26)^	Determine the incidence of post-COVID-19 symptoms in hospitalized patients and compare those in the Intensive Care Unit and those not in the Intensive Care Unit.	Cohort study, n=120	55% of men, with an average age of 54 years	Older adults (80-90 years old) presented with dry mouth, dysphagia, taste alterations, gingival bleeding, ulcers, and feeding difficulties. In addition, 3.3% reported persistent loss of taste, both in the Intensive Care Unit and non-Intensive Care Unit groups.
Patel D. *et al*., 2024, United Kingdom^(27)^	Investigate oral manifestations of long COVID and gather opinions and practices of healthcare professionals regarding these conditions.	Cross-sectional study, n=104	89.4% of women, aged between 18 and 79 years	47.1% reported oral symptoms lasting > three months: taste/smell alterations (58%), dry mouth (48.1%), and ulcers (45.7%).
Louisa M. *et al*., 2024, Indonesia^(28)^	Assess the distribution and severity of periodontal diseases in COVID-19 survivors with and without long COVID.	Cross-sectional study, n=40	Patients aged between 21 and 66 years The study did not consider gender.	Long COVID was associated with greater severity of periodontal diseases, such as gingivitis and generalized periodontitis (70%).

In the set of studies analyzed, the most frequently reported oral manifestations in long COVID were ageusia/dysgeusia, present in varying intensities^(14-18,21,23-26)^, and xerostomia^(19,21,22,27)^. Oral ulcers and gingival bleeding have also been described^(19,21,22)^, as well as difficulty chewing^(21,22)^, changes in the tongue^(19,22)^, and cases of gingivitis and periodontitis^(28)^. Some studies have found an association between female sex, older age, prolonged hospitalizations, and the presence of respiratory diseases, with a higher risk of persistent oral disorders^(15,20,24,25)^. It should be emphasized that this association in females was confirmed by statistical analyses and is not solely due to the numerical predominance of this group in the samples^(15,16,24)^. Furthermore, the influence of socioeconomic factors was highlighted, with a higher prevalence of symptoms in vulnerable populations, suggesting an impact of inequalities in access to healthcare^(18,24-26)^.

## DISCUSSION

The findings of this review highlight a wide diversity of oral manifestations associated with long COVID, reinforcing the importance of monitoring patients recovering from acute COVID-19 infection. Among the most frequently reported oral disorders are ageusia, xerostomia, taste alterations, and oral ulcerations.

Age proved to be a relevant factor in the manifestation of oral disorders associated with long COVID. Individuals over 65 years of age are more predisposed to persistent oral manifestations, including loss of taste and xerostomia^(29)^. This increased susceptibility may be associated with pathophysiological mechanisms, such as immunosenescence, characterized by the progressive reduction in the efficiency of the immune system with aging, and “inflammaging”, defined as a persistent state of low-grade chronic inflammation that accompanies age and contributes to greater vulnerability to diseases^(30)^. Furthermore, the presence of comorbidities in this age group increases the risk of prolonged hospitalizations and the need for intensive care, factors also associated with the worsening of oral manifestations^(31)^.

Despite this, persistent oral manifestations have also been reported in young adults, mainly changes in taste and smell, which may result both from an immunological response characteristic of this age group, being potentially more intense or dysregulated, and from the direct effect of the viral load on the olfactory and gustatory tissues^(29)^. Furthermore, long COVID has shown a higher prevalence among women, which may be related to multifactorial factors such as a more pronounced inflammatory response, hormonal influence, and immunological differences. It is also possible that aspects related to hospitalizations, such as physical strain and psychological repercussions, contribute to this greater vulnerability^(16,24,27)^.

Among the oral manifestations, loss of taste stood out as one of the most common^(16,23,24)^. The persistence of these disorders for weeks or months suggests possible prolonged damage to both the taste receptors and the neural pathways responsible for taste perception^(32)^. This dysfunction may result from local inflammatory processes, viral cytotoxic action, or alterations in cell regeneration, resulting in conditions ranging from mild to severe and disabling, with a direct impact on eating, the pleasure of eating, and, consequently, the quality of life of individuals^(15)^.

Another frequent manifestation was xerostomia, probably associated with salivary gland dysfunction induced by the viral infection and persistent inflammatory processes^(21)^. Reduced salivary flow compromises the lubrication of the oral cavity and the protective function of saliva, favoring the emergence of opportunistic infections, as well as causing masticatory difficulties, dysphagia, and increased vulnerability to cavities and periodontal diseases, negatively impacting overall oral health^(33)^. Furthermore, other manifestations include ulcerations, gingival bleeding, and orofacial pain^(25)^, generally attributed to oxidative stress, immunosuppression, and oral dysbiosis^(34,35)^.

SARS-CoV-2 infection can promote significant dysbiosis of the oral microbiome, with a reduction in bacterial diversity and the abundance of commensal species, associated with an increase in opportunistic pathogens^(36,37)^. These changes may be related to prolonged inflammation, hyposalivation, and the worsening of oral lesions, suggesting that the microbiome may play a central role in the pathophysiology of long COVID. In this sense, microbial modulation emerges in this scenario as a therapeutic possibility^(38)^.

Dental manifestations, specifically those affecting the periodontium, such as gingivitis and periodontitis, were observed mainly in patients with comorbidities and prolonged hospitalizations^(28)^. These conditions appear to result from both inflammatory and immunological exacerbation and compromised oral hygiene in hospital settings, which favors colonization by periodontal pathogens^(39)^. Therefore, differentiating between oral manifestations (related to mucous membranes and functions, such as taste and xerostomia) and dental manifestations (linked to dental support structures) becomes relevant, as it allows for directing prevention strategies, early diagnosis, and specific management in each clinical context, contributing to a more comprehensive approach to long COVID.

Prolonged hospitalization and orotracheal intubation have been identified as additional risk factors for oral manifestations such as dysphagia, candidiasis, and tongue coating, regardless of the presence of long COVID^(40,41)^. Studies show that post-extubation dysphagia can affect about one-fifth of patients admitted to the ICU. Furthermore, these patients frequently present with tongue coating and colonization by *Candida albicans, Streptococcus parasanguinis* and *Streptococcus mitis* ^(42)^. These effects are amplified in situations of immunosuppression, polypharmacy, invasive devices, and compromised oral hygiene, which are considered typical conditions in critical care settings, in addition to favoring opportunistic infections and overall oral disease.

Recognizing that such changes can occur independently of long COVID is essential for implementing appropriate clinical oral care protocols, as well as expanding the focus on hospital oral care, with a view to minimizing complications during and after hospitalization.

In addition to clinical factors related to hospitalization and the use of invasive devices, social determinants of health also play a relevant role in the persistence and worsening of oral manifestations of long COVID. Socioeconomic issues influence the identification and evolution of long COVID, with lower educational levels and less access to healthcare services being associated with delayed diagnoses and less adequate clinical follow-up^(43,44)^. In socially vulnerable communities, a high burden of comorbidities, difficulty accessing dental care, and limited resources for maintaining oral hygiene can contribute to the persistence of symptoms and worsening of oral manifestations^(43-45)^. Thus, socioeconomic status acts as an indirect factor, mediated by unequal access to healthcare, inadequate management of pre-existing diseases, and increased exposure to social determinants that worsen the clinical condition.

In contrast to the risk factors previously described, vaccination against COVID-19 has proven to be an important protective strategy. Evidence indicates that, in addition to reducing the occurrence of severe forms of the disease, immunization also significantly decreases the likelihood of persistent symptoms. Vaccinated individuals have a lower risk of developing long COVID, especially after three or more doses, which is reflected in a direct impact on both the severity and duration of clinical manifestations^(46,47)^. In this way, vaccination can indirectly reduce the frequency and intensity of oral manifestations related to long COVID, such as xerostomia, dysgeusia, and mucosal lesions, reinforcing its essential role not only in controlling COVID-19 but also in mitigating functional repercussions that compromise oral health and quality of life.

Interestingly, among the 15 selected articles, three (20%) were conducted in India, and two (15%) in Iran, while the remaining articles came from different countries (one study per country). This predominance may reflect, at least in part, the accelerated growth of scientific production in health in these countries in recent decades. Data from the National Science Foundation show that, between 2010 and 2022, India increased its production in health sciences by more than 180%, while Iran registered a growth of over 250% in the same period^(48)^. It may also be related to the high population density and the intense impact of the pandemic in these countries, also motivating further local investigation^(49)^. Furthermore, this concentration may reflect structural factors related to oral health in these contexts. In India, for example, the prevalence of periodontal disease affects about 95% of the population, while access to dental services is unequal, with less than 2% of dentists serving 72% of the population, which is predominantly rural in this country, and most services occur in the private sector^(50,51)^.

In Iran, socioeconomic inequalities are manifested through significant fragmentation in the index of decayed, missing, and filled teeth, concentrated among the most vulnerable groups^(52,53)^. Finally, the possibility of methodological bias in the selection process cannot be ruled out, since search criteria and database availability may favor certain regions or languages. In any case, this finding highlights the importance of considering the geographical representation of studies, motivating the search for more diverse data in future research.

In summary, this review contributes to the literature by systematizing and distinguishing the oral and dental manifestations associated with long COVID, highlighting the diversity of disorders, related factors (such as age, sex, hospitalization, and social inequalities), and the need for multidisciplinary follow-up. By integrating previously dispersed findings, this study expands the understanding of the topic and provides support for both clinical practice and the planning of public health policies, reinforcing the essential role of the dental perspective in the comprehensive management of long COVID.

### Study limitations

Among the limitations of this study is the possibility of bias in the selection of included articles stands out, since the review depends on the availability and quality of already published evidence. Furthermore, the heterogeneity of the contexts and populations investigated may limit the direct applicability of the findings to specific realities. Nevertheless, the results offer relevant insights for future research and for improving healthcare practices, especially in the context of addressing the oral and dental manifestations of long COVID.

### Contributions to health and public policies

The findings of this review contribute to expanding knowledge about the long-term effects of COVID-19 on oral health, alerting professionals and managers to the importance of integrating dental care into strategies for monitoring long COVID. By highlighting recurrent oral manifestations among vulnerable groups, the study reinforces the need for public policies that promote qualified surveillance, training of healthcare teams, and increased access to dental services within the Brazilian Health System.

## CONCLUSIONS

The review showed that, in different contexts and populations, oral manifestations associated with long COVID have been reported, including changes in taste, xerostomia, mucosal lesions, gingival bleeding, tooth loss, and difficulties with chewing, swallowing, and eating. These manifestations have been described more frequently in older individuals, women, those with comorbidities, prolonged hospitalization, and those in vulnerable socioeconomic contexts.

This review highlights the persistence of significant gaps regarding the pathophysiological mechanisms involved, such as the direct impact of SARS-CoV-2 on oral tissues. Furthermore, the association of oral manifestations with the use of medications, especially antivirals, corticosteroids, antibiotics, anticoagulants, antihypertensives, and psychotropic drugs, as well as with clinical factors such as mechanical ventilation and hyposalivation, still require further investigation. Therefore, the need for studies that delve deeper into these mechanisms and support the development of clinical protocols for screening, diagnosis, and management of oral manifestations of long COVID is reinforced, with interdisciplinary guidelines and strategies for functional and sensory rehabilitation.

## Data Availability

The research data are available in a repository: https://osf.io/r6uz8.
