# Supplementary material for: Oral manifestations associated with long COVID: a scoping review
Source: Rev Bras Enferm. 2026 Jul 6;79:e20250198. doi: 10.1590/0034-7167-2025-0198 (PMC13336229; doi:10.1590/0034-7167-2025-0198)
Supplement: Translation

# Translation: Manifestações orais associados à COVID longa: revisão de escopo

**DOI:** 10.1590/0034-7167-2025-0198pt

**Authors:** Emylly Lopes Theodoro, Karina Marques Prediger, Marielle Aparecida Damacena, Camilly Victória da Silva, Rodrigo das Neves Cano, Silvia Carla da Silva Andre Uehara

**Affiliations:** I Universidade Federal de São Carlos. São Carlos, São Paulo, Brasil

**Keywords:** Manifestações Bucais, Manifestações Orais, COVID Longa, Sequela Após COVID-19 Aguda, Doença Periodontal.

## Abstract

**Objetivos:**

mapear as evidências científicas sobre manifestações orais originados durante a COVID longa.

**Métodos:**

trata-se de revisão de escopo baseada no método descrito pelo JBI. Artigos primários foram incluídos em português, inglês e espanhol, publicados entre março de 2020 e dezembro de 2024, nas bases de dados PubMed, *Web of Science*, Biblioteca Virtual em Saúde, Scopus, *Excerpta Medica dataBASE* e *Scientific Electronic Library Online*, realizando-se análise descritiva.

**Resultados:**

dos 15 estudos analisados, as manifestações orais de COVID longa mais frequentes foram alterações gustativas, xerostomia, dificuldade de mastigar, sangramento gengival, periodontite e alterações nos dentes.

**Conclusões:**

evidenciou-se associação da COVID longa a diversas manifestações orais, impactando a qualidade de vida dos pacientes. Fatores como idade, comorbidades e desigualdades sociais influenciam a persistência destas manifestações, com maior prevalência em mulheres. A atuação multiprofissional e diretrizes mais claras são essenciais para aprimorar o cuidado odontológico.

## INTRODUÇÃO

A COVID longa foi caracterizada pela Organização Mundial da Saúde como a manifestação de sintomas que surgem geralmente dentro de três meses após a infecção aguda da COVID-19, perdurando por pelo menos dois meses, e não podem ser explicados por outro diagnóstico⁽^1^⁾. No Brasil, foi adotado oficialmente o termo “condições pós-COVID”, definido como a persistência ou o surgimento de sinais, sintomas e/ou alterações clínicas a partir de quatro semanas após a infecção inicial, podendo variar ao longo do tempo, com possíveis recidivas e, em alguns casos, evolução para complicações graves⁽^2^⁾. Neste estudo, optou-se pela utilização do termo “COVID longa”, em conformidade com a terminologia predominante na literatura internacional.

Desde o início da pandemia, observou-se que a COVID-19 vai além de uma infecção respiratória aguda, afetando diferentes sistemas do corpo. Já na fase pós-aguda, os sintomas persistentes mais frequentemente relatados incluem fadiga, dispneia, dor torácica, mialgia, distúrbios cognitivos e alterações do sono, variando em frequência e intensidade entre os indivíduos⁽^3,4^⁾. Essas manifestações têm gerado impactos importantes na qualidade de vida e na capacidade funcional dos pacientes, mesmo muitos meses após a recuperação da infecção inicial⁽^5^⁾.

A diversidade de apresentações clínicas da COVID longa está associada a fatores como idade, sexo, presença de comorbidades, gravidade do quadro inicial, necessidade de hospitalização ou internação em Unidade de Terapia Intensiva (UTI), além do *status* vacinal⁽^6,7^⁾. Evidências sugerem que mulheres e indivíduos não vacinados apresentam maior risco de desenvolver sintomas persistentes⁽^8^⁾. A resposta imunológica exacerbada e as alterações inflamatórias prolongadas também têm sido apontadas como possíveis mecanismos fisiopatológicos que explicam essa condição multifatorial⁽^9^⁾.

Nesse cenário, a cavidade oral emerge como um sítio de interesse para investigação das manifestações clínicas da COVID longa. Tecidos como a mucosa oral, a língua e as glândulas salivares expressam altos níveis da enzima conversora de angiotensina 2, que atua como principal receptor para a entrada do vírus nas células humanas⁽^10^⁾. Essa característica biológica sugere que a boca pode ser não apenas uma porta de entrada do vírus, mas também uma região vulnerável a manifestações clínicas persistentes. Cabe destacar que o uso da terminologia “manifestações orais” foi adotado neste estudo por se tratar de transtornos da boca que acompanham doenças ou lesões que não sejam de origem bucal, como é o caso da COVID longa^(11)^.

Estudos sobre sintomas orais da COVID longa permanece fragmentado, com relatos isolados e ausência de revisões abrangentes que sistematizem os achados existentes. Assim, diante do impacto da COVID longa e da manifestação de sintomas orais nessa condição, torna-se essencial mapear e sintetizar as evidências disponíveis. A identificação dessas manifestações pode favorecer a implementação e melhoria de protocolos de acompanhamento multiprofissional desses pacientes.

## OBJETIVOS

Mapear as evidências científicas sobre manifestações orais associadas à COVID longa.

## MÉTODOS

### Aspectos éticos

Por se tratar de estudo de revisão, não foi necessário apreciação pelo Comitê de Ética em Pesquisa. Porém, foi assegurado que todos os estudos primários contidos nesta revisão estivessem adequados às normas éticas vigentes.

### Tipo de estudo

Trata-se de revisão de escopo, que foi realizada seguindo os passos metodológicos descritos pelo JBI^(12)^, seguindo as diretrizes da *Preferred Reporting Items for Systematic reviews and Meta-Analyses extension for Scoping Review* ^(13)^.

### Procedimento metodológico

Para construção da questão norteadora, foi aplicada a estratégia PCC, que representa uma mnemônica para P (População) (Pessoas com COVID longa), C (Conceito) (manifestações orais) e C (Contexto) (pandemia de COVID-19), a ser definida como: quais as principais manifestações orais apresentadas por pessoas com COVID longa?

Foram incluídos estudos primários publicados em português, inglês e espanhol, entre março de 2020 e dezembro de 2024. Foram excluídos artigos cujos títulos e resumos não se delimitaram dentro do objetivo da investigação, além de artigos de opinião, editoriais, revisões, teses, dissertações e revisões secundárias. A decisão se fundamenta no propósito de concentrar a revisão em estudos originais publicados em periódicos científicos revisados por pares, assegurando maior rigor metodológico, confiabilidade das evidências e homogeneidade das fontes analisadas. Adicionalmente, foram aplicados filtros de período de publicação, idioma e tipo de documento em cada base de dados, conforme disponibilidade das plataformas consultadas. Também, foram verificadas as listas de referências de todos os estudos encontrados.

O protocolo desta revisão de escopo foi registrado no *Open Science Framework* (OSF), sob DOI 10.17605/OSF.IO/R6UZ8, e disponível em: https://osf.io/r6uz8.

### Coleta, organização e análise dos dados

A busca dos artigos foi realizada nas bases de dados eletrônicas PubMed, *Web of Science*, Biblioteca Virtual em Saúde, Scopus, *Excerpta Medica dataBASE* e *Scientific Electronic Library Online* no dia 17 de janeiro de 2025. Inicialmente, foi realizada uma busca exploratória para identificar as diferentes formas de indexação do fenômeno nas bases (descritores controlados *Medical Subject Headings* (MeSH)/Descritores em Ciências da Saúde (DeCS) e palavras livres), de modo a contemplar variações terminológicas e sinonímias relevantes.

A condução das buscas ocorreu por meio de descritores e seus sinônimos, que constam no DeCS e no MeSH, sendo eles: *Long COVID; Post-Acute COVID-19 Syndrome; Post-COVID Conditions; Signs and Symptoms; Oral Manifestations; Periodontal Diseases; Parodontosis; Periodontal Atrophy; Gingivo-Osseous Atrophies; Stomatognathic Diseases; Dental Diseases; Mouth and Tooth Diseases; Pathology, Oral; Oral and Maxillofacial Pathology; Tooth Loss*. A combinação dos descritores foi realizada através do uso dos booleanos *OR* e *AND* (Quadro 1).

**Quadro 1:** Estratégias de busca utilizadas nas bases de dados, São Carlos, São Paulo, Brasil, 2025

Base de dados	Referência	Total de artigos
PubMed	*((((((((((((((“Long COVID”[Title]) OR “Post-Acute COVID-19 Syndrome”[Title]) OR “Post-COVID Conditions”[Title]) AND (“Signs[Title] AND Symptoms”[Title])) OR “Oral Manifestations”[Title]) OR “Periodontal Diseases”[Title]) OR “Parodontosis”[Title]) OR “Periodontal Atrophy”[Title]) OR “Gingivo-Osseous Atrophies”[Title]) OR “Stomatognathic Diseases”[Title]) OR “Dental Diseases”[Title]) OR (“Mouth[Title] AND Tooth Diseases”[Title])) OR “Pathology, Oral”[Title]) OR (“Oral[Title] AND Maxillofacial Pathology”[Title])) OR “Tooth Loss”[Title]*	573
Biblioteca Virtual em Saúde	*(ab:(“Long COVID” OR “Post-Acute COVID-19 Syndrome” OR “Post-COVID Conditions” OR “*Síndrome de COVID-19 Pós-Aguda*” OR “Síndrome Post Agudo de COVID-19”)) AND (ab:(“Signs and Symptoms” OR “*Sinais e Sintomas*” OR “Signos y Síntomas”)) AND (ab:(“Oral Manifestations” OR “*Manifestações Bucais*” OR “Manifestaciones Bucales”)) OR (ab:(“Periodontal Diseases” OR Parodontosis OR “*Doenças Periodontais*” OR “Enfermedades Periodontales”)) OR (ab:(“Periodontal Atrophy” OR “Gingivo-Osseous Atrophies” OR “*Atrofia Periodontal*”)) OR (ab:(“Stomatognathic Diseases” OR “Dental Diseases” OR “Mouth and Tooth Diseases” OR “*Doenças Estomatognáticas*” OR “Enfermedades Estomatognáticas”)) OR (ab:(“Pathology, Oral” OR “Oral and Maxillofacial Pathology” OR “*Patologia Bucal*” OR “Patología Bucal”)) OR (ab:(“Tooth Loss” OR “*Perda de Dente*” OR “Pérdida de Diente”))*	95
Scopus	*( ABS ( “Long COVID” ) OR ABS ( “Post-Acute COVID-19 Syndrome” ) OR ABS ( “Post-COVID Conditions” ) AND ABS ( “Signs and Symptoms” ) OR ABS ( “Oral Manifestations” ) OR ABS ( “Periodontal Diseases” ) OR ABS ( parodontosis ) OR ABS ( “Periodontal Atrophy” ) OR ABS ( “Gingivo-Osseous Atrophies” ) OR ABS ( “Stomatognathic Diseases” ) OR ABS ( “Dental Diseases” ) OR ABS ( “Mouth and Tooth Diseases” ) OR ABS ( “Pathology, Oral” ) OR ABS ( “Oral and Maxillofacial Pathology” ) OR ABS ( “Tooth Loss” ) )*	95
*Scientific Electronic Library Onlin*e	POR TÍTULO: (ti:(^*^”Long COVID” OR “Post-Acute COVID-19 Syndrome” OR “Post-COVID Conditions” OR “Síndrome de COVID-19 Pós-Aguda*” OR “Síndrome Post Agudo de COVID-19” )) AND (ti:(“Signs and Symptoms” OR “*Sinais e Sintomas*” OR “Signos y Síntomas” )) AND (ti:(“Oral Manifestations” OR “*Manifestações Bucais*” OR “Manifestaciones Bucales” )) OR (ti:(“Periodontal Diseases” OR Parodontosis OR “*Doenças Periodontais*” OR “Enfermedades Periodontales” )) OR (ti:(“Periodontal Atrophy” OR “Gingivo-Osseous Atrophies” OR “*Atrofia Periodontal*” )) OR (ti:(“Stomatognathic Diseases” OR “Dental Diseases” OR “Mouth and Tooth Diseases” OR “*Doenças Estomatognáticas*” OR “Enfermedades Estomatognáticas” )) OR (ti:(“Pathology, Oral” OR “Oral and Maxillofacial Pathology” OR “*Patologia Bucal*” OR “Patología Bucal”)) OR (ti:(“Tooth Loss” OR “*Perda de Dente*” OR “Pérdida de Diente”))*	40
*Web of Science*	*(((((((TI=(“Long COVID” OR “Post-Acute COVID-19 Syndrome” OR “Post-COVID Conditions”)) AND TI=(“Signs and Symptoms”)) AND TI=(“Oral Manifestations”)) OR TI=(“Periodontal Diseases” OR “Parodontosis”)) OR TI=(“Periodontal Atrophy” OR “Gingivo-Osseous Atrophies” )) OR TI=(“Stomatognathic Diseases” OR “Dental Diseases” OR “Mouth and Tooth Diseases”)) OR TI=(“Pathology, Oral” OR “Oral and Maxillofacial Pathology”)) OR TI=(“Tooth Loss”)*	888
*Excerpta Medica dataBASE*	*((‘long Covid’:ti OR ‘post-acute Covid-19 syndrome’:ti OR ‘post-Covid conditions’:ti) AND ‘signs and symptoms’:ti AND ‘oral manifestations’:ti OR ‘periodontal diseases’:ti OR ‘parodontosis’:ti OR ‘periodontal atrophy’:ti OR ‘gingivo-osseous atrophies’:ti OR ‘stomatognathic diseases’:ti OR ‘dental diseases’:ti OR ‘mouth and tooth diseases’:ti OR ‘pathology, oral’:ti OR ‘oral and maxillofacial pathology’:ti OR ‘tooth loss’:ti) AND [2020-2024]/py*	927

Para seleção dos estudos, após implementação da estratégia de busca em cada base de dados, as referências foram exportadas para o aplicativo web StArt (*State of the Art through Systematic Review*) para seleção dos estudos em dois níveis. Nesta etapa, foram realizadas a exclusão de duplicações de forma automática pelo *software* e, posteriormente, a verificação manual para confirmação. A primeira seleção foi realizada por meio da leitura de títulos e resumos, seguida pela leitura do artigo na íntegra. A ferramenta de revisão StArt foi desenvolvida pelo Laboratório de Pesquisa em Engenharia de *Software* da Universidade Federal de São Carlos.

Os estudos elegíveis foram avaliados na íntegra por dois pesquisadores individualmente, e as possíveis divergências seriam discutidas com um terceiro pesquisador e solucionadas por consenso. Entretanto, não foi necessário acionar um terceiro pesquisador, pois não houve divergências. Foram extraídos dados de cada artigo incluído, como autores, país de realização do estudo, data de publicação, objetivo da pesquisa, desenho do estudo e principais resultados. Para apresentação dos resultados, foram utilizados quadros e descrições, de forma a responder à pergunta norteadora da revisão.

## RESULTADOS

Foram encontrados 2.618 artigos nas bases de dados, dos quais 1.253 foram removidos por serem duplicados. Entre os 1.365 estudos analisados, foram excluídos 388, por serem artigos de opinião, editoriais, revisões, manuais, teses ou dissertações, de acordo com os critérios de exclusão previamente estabelecidos, e 960 após leitura de título e resumo. Assim, 17 foram lidos na íntegra, sendo oito excluídos por não responderem à pergunta norteadora, e nove, selecionados para o estudo. Analisando as referências dos nove artigos selecionados, foram incluídos seis estudos a partir das referências citadas, por abordarem o tema proposto, totalizando 15 estudos nesta revisão (Figura 1).

**Figura 1:** Fluxograma de seleção de artigos, São Carlos, São Paulo, Brasil, 2025

Entre os 15 artigos incluídos, três (20%) foram conduzidos na Índia, dois (13,3%), no Irã, e um (6,7%), em cada um dos seguintes países: Suécia, Polônia, Reino Unido, Iraque, Arábia Saudita, Brasil, França, Espanha, Indonésia e Ilhas Faroé. Observa-se que 14 (93,3%) artigos foram publicados em inglês, e apenas um (6,7%), em português.

Em relação ao delineamento, seis (40%) eram estudos transversais, cinco (33,3%), de coorte, dois (13,3%), retrospectivos, um (6,7%), longitudinal, e um (6,7%), relato de caso.

Os estudos incluídos foram descritos quanto ao objetivo, tipo de delineamento, caracterização da população do estudo e principais evidências encontradas. Destaca-se que, na fase pós-aguda da infecção por SARS-CoV-2, foram relatadas uma ou mais manifestações orais, incluindo manifestações odontológicas, quando afetam estruturas periodontais, conforme sistematizado no Quadro 2.

**Quadro 2:** Descrição dos artigos segundo autor, ano, local, objetivo, tipo de estudo, amostra e principais resultados, São Carlos, São Paulo, Brasil, 2025

Autor, ano e local	Objetivo	Tipo de estudo e amostra (n)	Caracterização da população do estudo	Principais evidências encontradas
Moradian S.T. *et al*., 2020, Irã^(14)^	Avaliar a frequência de sintomas tardios em pacientes com COVID-19.	Estudo transversal, n=200	80% de homens, com idade média de 55 anos	Distúrbios persistentes do paladar relatados em 8% após seis semanas da alta hospitalar, indicando sequelas gustativas da COVID longa.
Asadi-Pooya A.A. *et al*., 2021, Irã^(15)^	Examinar fatores de risco associados ao desenvolvimento de COVID longa em uma grande coorte.	Estudo retrospectivo, n=4.681	52,9% de homens, com idade média de 52 anos	Perda de paladar em 3% dos casos, mais frequente em mulheres, pessoas com doenças respiratórias e internações prolongadas.
Zayet S. *et al*., 2021, França^(16)^	Descrever pacientes com síndrome pós-COVID-19 com sintomas persistentes e compará-los ao grupo controle.	Estudo retrospectivo, n=354	63% de mulheres, com idade entre 19 e 98 anos	31,5% relataram perda de paladar após cerca de nove meses da infecção, reforçando a persistência de sintomas gustativos.
Riestra-Ayora J. *et al*., 2021, Espanha^(17)^	Determinar a incidência e evolução de disfunções olfativas e gustativas relacionadas à COVID-19.	Estudo de coorte, n=320	81,9% de mulheres, com idade entre 18 e 65 anos 60,5% apresentaram sintomas gustativos persistentes.	60,5% apresentaram sintomas gustativos persistentes, sendo que 12% não tiveram recuperação, e 14,4% tiveram recuperação parcial após seis meses.
Petersen M.S. *et al*., 2021, Ilhas Faroé^(18)^	Descrever sintomas agudos e de COVID longa em pacientes não hospitalizados.	Estudo longitudinal, n=180	54% de mulheres, com idade média de 39,9 anos	Perda de paladar em 16,4% na fase aguda, persistindo em 1,7% no seguimento tardio em não hospitalizados.
Rafałowicz B. *et al*., 2022, Polônia^(19)^	Apresentar, por meio de seis casos, os sintomas orais mais comuns de COVID longa em pacientes com histórico de infecção por SARS-CoV-2.	Relato de caso, n=6	Quatro homens (66%) e duas mulheres (34%) entre 43 e 72 anos	Casos clínicos evidenciaram boca seca, lesões orais, perda de dentes e alterações de língua associadas à COVID longa.
Alfadda A.A. *et al*., 2022, Arábia Saudita^(20)^	Investigar características clínicas e bioquímicas de pacientes após seis meses de alta hospitalar.	Estudo de coorte, n=98	51 (52%) homens, com idade média de 48 anos	Perda de paladar e apetite relatada por 7,1% após seis meses da alta hospitalar.
Muthyam A.K. *et al*., 2022, Índia^(21)^	Determinar manifestações orais entre pacientes recuperados de COVID-19.	Estudo transversal, n=100	51% de homens e 54% de mulheres >35 anos	Xerostomia (11%), ulcerações (12%), alterações do paladar (11%), sangramento gengival e dificuldades mastigatórias identificados.
Johansson A.K. *et al*., 2023, Suécia^(22)^	Fornecer informações sobre sintomas gerais e orofaciais da COVID-19 aguda e longa em sobreviventes suecos, e identificar fatores preditivos/protetores.	Estudo de coorte, n=283	132 pacientes com COVID longa, 60 mulheres (45%) e 72 homens (55%) entre 80 e 90 anos	46% apresentaram sintomas orais, como dificuldade mastigatória, dentes rachados, boca seca, dificuldades alimentares, sangramento gengival, disfagia, alterações no paladar e úlceras. Além disso, 14% tiveram ≥5 sintomas. Houve ageusia persistente em até seis meses após a recuperação, embora em proporções decrescentes ao longo do tempo.
Al-Magsoosi M.J.N. *et al*., 2023, Iraque^(23)^	Determinar a prevalência de manifestações orais de COVID-19 em pacientes recuperados.	Estudo transversal, n=574	378 (88,3%) mulheres e 196 (11,7%) homens, com idade entre 18 e 78 anos	Manifestações orais ocorreram em 88,3%, principalmente ageusia, xerostomia e disfagia. Apenas a ageusia persistiu, com associação à gravidade e idade, sem relação com sexo, tabagismo ou comorbidades.
Araújo A.D.D.G. *et al*., 2023, Brasil^(24)^	Analisar fatores relacionados à COVID longa na população adulta do Brasil.	Estudo transversal analítico, n=288	71,9% de mulheres, com idade entre 18 e 91 anos	As manifestações clínicas associadas à persistência foram febre, calafrios, dor de cabeça, tosse, anosmia, ageusia, dispneia e diarreia. Entre os persistentes, prevaleceram dor de cabeça (24,65%), tosse (21,88%) e febre (21,18%), enquanto diarreia e dispneia tiveram menor frequência (13,19%).
Gujral H.S. *et al*., 2023, Índia^(25)^	Estudar efeitos posteriores até 12 meses após COVID-19 aguda e analisar preditores de risco.	Estudo de coorte, n=120	63,3% de homens, com idade média de 41 anos	3,3% apresentaram perda de paladar persistente após hospitalização, independentemente de internação em Unidade de Terapia Intensiva.
Nair C.V. *et al*., 2023, Índia^(26)^	Determinar incidência de sintomas pós-COVID-19 em hospitalizados e comparar Unidade de Terapia Intensiva e não Unidade de Terapia Intensiva.	Estudo de coorte, n=120	55% de homens, com idade média de 54 anos	Idosos (80-90 anos) apresentaram boca seca, disfagia, alterações no paladar, sangramento gengival, úlceras e dificuldades alimentares. Além disso, 3,3% relataram perda de paladar persistente, tanto em grupo na Unidade de Terapia Intensiva quanto na não Unidade de Terapia Intensiva.
Patel D. *et al*., 2024, Reino Unido^(27)^	Investigar manifestações orais da COVID longa e levantar opiniões e práticas de profissionais de saúde sobre essas condições.	Estudo transversal, n=104	89,4% de mulheres, com idade entre 18 e 79 anos	47,1% relataram sintomas orais > três meses: alterações no paladar/olfato (58%), boca seca (48,1%) e úlceras (45,7%).
Louisa M. *et al*., 2024, Indonésia^(28)^	Avaliar a distribuição e gravidade de doenças periodontais em sobreviventes de COVID-19 com e sem COVID longa.	Estudo transversal, n=40	Paciente com idade entre 21 e 66 anos O estudo não relacionou o sexo.	COVID longa associada foi a maior gravidade de doenças periodontais, como gengivite e periodontite generalizada (70%).

No conjunto dos estudos analisados, as manifestações orais mais frequentemente relatadas na COVID longa foram ageusia/disgeusia, presente em diferentes intensidades^(14-18,21,23-26)^ e xerostomia^(19,21,22,27)^. Também foram descritas úlceras orais e sangramento gengival^(19,21,22)^, dificuldade mastigatória^(21,22)^, alterações na língua^(19,22)^, além de quadros de gengivite e periodontite^(28)^. Em alguns estudos, verificou-se associação entre sexo feminino, idade mais avançada, internações prolongadas e presença de doenças respiratórias, com maior risco para persistência de transtornos orais^(15,20,24,25)^. Ressalta-se que essa associação no sexo feminino foi confirmada por análises estatísticas, não se devendo apenas ao predomínio numérico desse grupo nas amostras^(15,16,24)^. Ademais, destacou-se a influência de fatores socioeconômicos, com maior prevalência de sintomas em populações em situação de vulnerabilidade, sugerindo impacto das desigualdades no acesso ao cuidado em saúde^(18,24-26)^.

## DISCUSSÃO

Os achados desta revisão evidenciam uma ampla diversidade de manifestações orais associados à COVID longa, reforçando a importância do acompanhamento dos pacientes em recuperação da infecção aguda pela COVID-19. Entre os transtornos da boca mais frequentemente relatados, destacam-se ageusia, xerostomia, alterações no paladar e ulcerações orais.

A idade mostrou-se um fator relevante na manifestação de transtornos orais associados à COVID longa. Indivíduos com mais de 65 anos apresentam maior predisposição para manifestações orais persistentes, incluindo perda de paladar e xerostomia^(29)^. Essa maior suscetibilidade pode estar associada a mecanismos fisiopatológicos, como a imunossenescência, caracterizada pela redução progressiva da eficiência do sistema imunológico com o envelhecimento, e o “*inflammaging*”, definido como um estado persistente de inflamação crônica de baixo grau que acompanha a idade e contribui para maior vulnerabilidade às doenças^(30)^. Além disso, a presença de comorbidades nessa faixa etária aumenta o risco de hospitalizações prolongadas e necessidade de UTI, fatores também associados ao agravamento das manifestações orais^(31)^.

Apesar disso, manifestações orais persistentes também foram relatadas em adultos jovens, principalmente alterações de paladar e olfato, que podem decorrer tanto de uma resposta imunológica característica dessa faixa etária, sendo potencialmente mais intensa ou desregulada, quanto do efeito direto da carga viral sobre os tecidos olfatórios e gustativos^(29)^. Além disso, a COVID longa mostrou maior prevalência entre mulheres, o que pode estar relacionado a fatores multifatoriais, como resposta inflamatória mais acentuada, influência hormonal e diferenças imunológicas. Também é possível que aspectos ligados às hospitalizações, como sobrecarga física e repercussões psicológicas, contribuam para essa maior vulnerabilidade^(16,24,27)^.

Entre as manifestações orais, a perda do paladar se destacou como uma das manifestações mais comuns^(16,23,24)^. A persistência desses transtornos por semanas ou meses sugere um possível dano prolongado tanto aos receptores gustativos quanto às vias neurais responsáveis pela percepção do paladar^(32)^. Essa disfunção pode decorrer de processos inflamatórios locais, ação citotóxica viral ou alterações na regeneração celular, resultando em quadros que variam de leves a graves e incapacitantes, com impacto direto na alimentação, no prazer de comer e, consequentemente, na qualidade de vida dos indivíduos^(15)^.

Outra manifestação frequente foi a xerostomia, provavelmente associada à disfunção das glândulas salivares induzida pela infecção viral e por processos inflamatórios persistentes^(21)^. A redução do fluxo salivar compromete a lubrificação da cavidade oral e a função protetora da saliva, favorecendo o surgimento de infecções oportunistas, além de gerar dificuldades mastigatórias, disfagia, e maior vulnerabilidade a cáries e doenças periodontais, repercutindo negativamente na saúde bucal como um todo^(33)^. Ainda, outras manifestações incluem ulcerações, sangramento gengival e dor orofacial⁽^2 5^⁾, geralmente atribuídos ao estresse oxidativo, imunossupressão e disbiose oral^(34,35)^.

A infecção por SARS-CoV-2 pode promover disbiose significativa do microbioma oral, com redução da diversidade bacteriana e da abundância de espécies comensais, associada ao aumento de patógenos oportunistas^(36,37)^. Essas alterações podem estar relacionadas à inflamação prolongada, à hipossalivação e ao agravamento de lesões orais, sugerindo que o microbioma pode desempenhar papel central na fisiopatologia da COVID longa. Nesse sentido, a modulação microbiana surge neste cenário como uma possibilidade terapêutica^(38)^.

Já as manifestações odontológicas, aquelas que acometem o periodonto, como gengivite e periodontite, foram observadas principalmente em pacientes com comorbidades e hospitalizações prolongadas^(28)^. Esses quadros parecem decorrer tanto da exacerbação inflamatória e imunológica quanto da higiene oral comprometida em ambientes hospitalares, o que favorece a colonização por patógenos periodontais^(39)^. Assim, a diferenciação de manifestações orais (relacionadas a mucosas e funções, como paladar e xerostomia) das odontológicas (ligadas às estruturas de suporte dentário) se torna relevante, pois permite direcionar estratégias de prevenção, diagnóstico precoce e manejo específico em cada contexto clínico, contribuindo para uma abordagem mais abrangente da COVID longa.

A hospitalização prolongada e a intubação orotraqueal têm se configurado como fatores de risco adicionais para manifestações orais como disfagia, candidíase e saburra lingual, independentemente da presença de COVID longa^(40,41)^. Estudos mostram que a disfagia pós-extubação pode afetar cerca de um quinto dos pacientes internados em UTI. Além disso, esses pacientes frequentemente apresentam saburra lingual com colonização por *Candida albicans, Streptococcus parasanguinis* e *Streptococcus mitis* ^(42)^. Esses efeitos são potencializados em situações de imunossupressão, polimedicação, dispositivos invasivos e higiene oral comprometida, sendo consideradas condições típicas de internações críticas, além de favorecer infecções oportunistas e adoecimento bucal global.

Reconhecer que tais alterações podem ocorrer independentemente da COVID longa é essencial para implementação de protocolos de cuidados orais clínicos adequados, além da ampliação do foco na atenção bucal hospitalar, com vistas a minimizar as complicações durante e após a hospitalização.

Além dos fatores clínicos relacionados à hospitalização e ao uso de dispositivos invasivos, os determinantes sociais de saúde também desempenham papel relevante na persistência e agravamento das manifestações orais da COVID longa. Questões socioeconômicas exercem influência sobre a identificação e evolução da COVID longa, sendo que níveis educacionais mais baixos e menor acesso aos serviços de saúde estão associados a diagnósticos tardios e menor acompanhamento clínico adequado^(43,44)^. Em comunidades socialmente vulneráveis, sobrecarga de comorbidades, dificuldade de acesso a cuidados odontológicos e limitação de recursos para manutenção da higiene bucal podem favorecer a persistência de sintomas e piora de manifestações orais^(43-45)^. Dessa forma, a condição socioeconômica atua como um fator indireto, mediado pelo acesso desigual à saúde, pelo manejo inadequado de doenças pré-existentes e pelo aumento da exposição a determinantes sociais que agravam o quadro clínico.

Em contrapartida aos fatores de risco previamente descritos, a vacinação contra a COVID-19 tem se mostrado uma importante estratégia protetora. Evidências apontam que, além de reduzir a ocorrência de formas graves da doença, a imunização também diminui significativamente a probabilidade de manifestações persistentes. Indivíduos vacinados apresentam menor risco de desenvolver COVID longa, especialmente após três ou mais doses, o que se reflete em impacto direto tanto na gravidade quanto na duração das manifestações clínicas^(46,47)^. Dessa forma, a vacinação pode atuar indiretamente na redução da frequência e da intensidade das manifestações orais relacionadas à COVID longa, como xerostomia, disgeusia e lesões em mucosa, reforçando seu papel essencial não apenas no controle da COVID-19, mas também na mitigação de repercussões funcionais que comprometem a saúde bucal e qualidade de vida.

Curiosamente, entre os 15 artigos selecionados, três (20%) foram conduzidos na Índia, e dois (15%), no Irã, enquanto os restantes provêm de diferentes países (um estudo por país). Esse predomínio pode refletir, pelo menos em parte, o acelerado crescimento da produção científica em saúde nesses países nas últimas décadas. Dados da *National Science Foundation* mostram que, entre 2010 e 2022, a Índia aumentou sua produção em ciências da saúde em mais de 180%, enquanto o Irã registrou um crescimento superior a 250% nesse mesmo período^(48)^. Também, pode estar relacionado à elevada carga populacional e ao impacto intenso da pandemia nesses países, motivando também maior investigação local^(49)^. Além disso, essa concentração pode refletir fatores estruturais relacionados à saúde bucal nesses contextos. Na Índia, por exemplo, a prevalência de doenças periodontais afeta cerca de 95% da população, ao passo que o acesso aos serviços odontológicos é desigual, sendo que menos de 2% dos dentistas atendem 72% da população, que é predominante rural neste país, e a maioria dos atendimentos ocorre no setor privado^(50,51)^.

No Irã, desigualdades socioeconômicas se manifestam por meio de fragmentação significativa no índice de dentes cariados, perdidos e obturados, concentrado entre os grupos mais vulneráveis^(52,53)^. Por fim, não se pode descartar a possibilidade de viés metodológico no processo de seleção, visto que critérios de busca e disponibilidade de bases podem favorecer determinadas regiões ou línguas. De toda forma, esse achado ressalta a importância de considerar a representação geográfica dos estudos, motivando a busca por dados mais diversificados em futuras pesquisas.

Em síntese, a presente revisão contribui para a literatura ao sistematizar e distinguir as manifestações orais e odontológicas associadas à COVID longa, destacando a diversidade de transtornos, os fatores relacionados (como idade, sexo, hospitalização e desigualdades sociais) e a necessidade de acompanhamento multiprofissional. Ao integrar achados previamente dispersos, este estudo amplia a compreensão sobre o tema e fornece subsídios tanto para a prática clínica quanto para o planejamento de políticas públicas em saúde, reforçando o papel essencial do olhar odontológico no manejo integral da COVID longa.

### Limitações do estudo

Entre as limitações deste estudo, destaca-se a possibilidade de viés na seleção dos artigos incluídos, uma vez que a revisão depende da disponibilidade e qualidade das evidências já publicadas. Além disso, a heterogeneidade dos contextos e populações investigadas pode limitar a aplicabilidade direta dos achados em determinadas realidades. Ainda assim, os resultados oferecem subsídios relevantes para futuras pesquisas e para o aprimoramento das práticas em saúde, especialmente no contexto da atenção às manifestações orais e odontológicas da COVID longa.

### Contribuições para as áreas da saúde e políticas públicas

Os achados desta revisão contribuem para ampliar o conhecimento sobre os efeitos de longo prazo da COVID-19 na saúde bucal, alertando profissionais e gestores para a importância de integrar o cuidado odontológico nas estratégias de acompanhamento da COVID longa. Ao evidenciar manifestações orais recorrentes entre grupos vulneráveis, o estudo reforça a necessidade de políticas públicas que promovam vigilância qualificada, capacitação das equipes de saúde e ampliação do acesso aos serviços odontológicos no Sistema Único de Saúde.

## CONCLUSÕES

A revisão evidenciou que, em diferentes contextos e populações, foram relatadas manifestações orais associadas à COVID longa, incluindo alterações no paladar, xerostomia, lesões de mucosa, sangramentos gengivais, perda dentária, e dificuldades de mastigação, deglutição e alimentação. Essas manifestações têm sido descritos com maior frequência em indivíduos mais idosos, do sexo feminino, com comorbidades, hospitalização prolongada e inseridos em contextos socioeconômicos vulneráveis.

Esta revisão mostra a persistência de lacunas relevantes sobre os mecanismos fisiopatológicos envolvidos, como o impacto direto do SARS-CoV-2 nos tecidos orais. Além disso, a associação de manifestações orais com o uso de medicamentos, especialmente antivirais, corticóides, antibióticos, anticoagulantes, anti-hipertensivos e psicofármacos, bem como com fatores clínicos como ventilação mecânica e hipossalivação, ainda exigem maior investigação. Assim, reforça-se a necessidade de estudos que aprofundem esses mecanismos e subsidiem a elaboração de protocolos clínicos para rastreamento, diagnóstico e manejo das manifestações orais da COVID longa, com diretrizes interdisciplinares e estratégias de reabilitação funcional e sensorial.

## Data Availability

Os dados de pesquisa estão disponíveis em repositório: https://osf.io/r6uz8.
